# Structural and functional basis for starch binding in the SnRK1 subunits AKINβ2 and AKINβγ

**DOI:** 10.3389/fpls.2014.00199

**Published:** 2014-05-16

**Authors:** Alejandra Ávila-Castañeda, Natalia Gutiérrez-Granados, Ana Ruiz-Gayosso, Alejandro Sosa-Peinado, Eleazar Martínez-Barajas, Patricia Coello

**Affiliations:** ^1^Departamento de Bioquímica, Facultad de Química, Universidad Nacional Autónoma de MéxicoMéxico City, México; ^2^Departamento de Bioquímica, Facultad de Medicina, Universidad Nacional Autónoma de MéxicoMéxico City, México

**Keywords:** Starch Binding Domain (SBD), SnRK1, AKINβγ, AKINβ2, chloroplast proteins

## Abstract

Specialized carbohydrate-binding domains, the Starch-Binding Domain (SBD) and the Glycogen Binding Domain (GBD), are motifs of approximately 100 amino acids directly or indirectly associated with starch or glycogen metabolism. Members of the regulatory β subunit of the heterotrimeric complex AMPK/SNF1/SnRK1 contain an SBD or GBD. In *Arabidopsis thaliana*, the β regulatory subunit AKINβ2 and a γ-type subunit, AKINβγ, also have an SBD. In this work, we compared the SBD of AKINβ2 and AKINβγ with the GBD present in rat AMPKβ1 and demonstrated that they conserved the same overall topology. The majority of the amino acids identified in the protein-carbohydrate interactions in the rat AMPKβ1 are conserved in the two plant proteins. In AKINβγ, there is an insertion of three amino acids that creates a loop adjacent to one of the conserved tryptophan residues. Functionally, the SBD from AKINβγ and AKINβ2 could bind starch, but there was an important difference in the association when an amylose/amylopectin (A/A) mixture was used. The physiological relevance of binding to starch was clear for AKINβγ, because immunolocalization experiments identified this protein inside the chloroplast. SnRK1 activity was not affected by the addition of A/A to the reaction mixture. However, addition of starch inhibited the activity 85%. Furthermore, proteins associated with A/A and starch in an *in vitro*-binding assay accounted for 10–20% of total SnRK1 kinase activity. Interestingly, the identification of the SnRK1 subunits associated to the protein-carbohydrate complex indicated that only the catalytic subunits, AKIN10 and AKIN11, and the regulatory subunit AKINβγ were present. These results suggest that a dimer formed between either catalytic subunit and AKINβγ could be associated with the A/A mixture in its active form but the same subunits are inactivated when binding to starch.

## Introduction

Arabidopsis SnRK1 is a protein kinase involved in cellular signaling in response to low-energy stress and carbon status (Baena-González et al., 2007; Fragoso et al., 2009; Coello et al., 2011; Nunes et al., 2013a,b). It also functions in hormonal and stress responses and during growth and development (Radchuk et al., 2006; Baena-González and Sheen, 2008; Martínez-Barajas et al., 2011; Cho et al., 2012; Nunes et al., 2013a). SnRK1 is a heterotrimeric complex formed by an α catalytic subunit and two regulatory β and γ subunits. In Arabidopsis, there are two isoforms of the catalytic subunit (AKIN10 and AKIN11), three β subunits (AKINβ1, AKINβ2, and AKINβ3) and one γ subunit (AKINg). Furthermore, there is a plant-specific protein, AKINβγ, that contains a sequence at the N-terminus that is homologous to the β subunits and four motifs at the C-terminus homologous to the γ subunits (Lumbreras et al., 2001; Gissot et al., 2006; Polge and Thomas, 2007; Ramon et al., 2013). All subunits that form a part of the complex are well conserved between mammals, yeast and plants. Sequence alignments of the β subunits have shown conserved domains in the different organisms. These domains include the Association with the SNF1 Complex (ASC) domain, which is important for interaction with the α and γ subunits, and a specialized carbohydrate-binding module named the Glycogen Binding Domain (GBD), because it facilitates the association of the protein with glycogen in animals and yeast (Ghillebert et al., 2011; Sanz et al., 2013). In Arabidopsis, only AKINβ1 and AKINβ2 exhibited both the GBD and the ASC domain (Gissot et al., 2004; Polge et al., 2008), AKINβ 3 does not have a GBD and only conserved the ASC domain. Interestingly, AKINβγ also has an N-terminal homology to the GBD, despite functional characterization as a γ subunit and not a β subunit (Lumbreras et al., 2001). The GBD in the β subunits was proposed to be a regulatory domain that inhibits AMPK activity when it was bound to the branching points within glycogen, acting as a glycogen sensor in mammalian cells (McBride et al., 2009). It might also be important to the localization of the complex near putative targets that participate in glycogen metabolism, such as glycogen synthase and glycogen phosphorylase (Hudson et al., 2003; Polekhina et al., 2003). To understand the GBD-glycogen association, a 3D atomic structure of AMPKβ 1-GBD in complex with β-cyclodextrin was obtained (Polekhina et al., 2005). The structure reveals W100, K126, W133, and N150 as critical residues for protein-carbohydrate interaction. These residues have been confirmed by mutational analysis that resulted in the disruption of GBD-glycogen association (Polekhina et al., 2003, 2005). In yeast, the role of glycogen in the regulation of SNF1 is not clear, because blocking glycogen synthesis had no impact on its activity. In addition, deletion of the GBD in Gal83, one of the β subunits, increased the activity of SNF1 and released the yeast from glucose repression, suggesting that it acts as a negative regulator (Mangat et al., 2010). The role of the GBD in the plant subunits AKINβ and AKINβγ has not been evaluated and their ability to bind starch, the plant carbohydrate storage molecule, is unknown. Glycogen and starch are the main storage carbohydrates found in living cells, made of α-1, 4 linked chains of glucose that are branched together through α-1, 6 linkages. Glycogen is a homogeneous water-soluble polymer with a particle size of less than 50 nm of diameter. Thus glycogen particles are accessible to a rapid mobilization by enzymes of the glycogen metabolism (Ball and Morell, 2003). Unlike glycogen, starch is found as granules that have a semi-crystalline structure and unlimited size making them unavailable to hydrosoluble enzymes. Starch is composed of a mixture of two different polysaccharides: the major fraction is the amylopectin that accounts for 70–90% of the granule weight of most starches. It forms a molecule consisting of 100,000–1,000,000 glucosyl units. In contrast, amylose is a smaller molecule having around 1000 glucosyl units with fewer branch points (Streb and Zeeman, 2012; Cenci et al., 2014). The differences between glycogen and starch might imply important changes in the recognition by regulatory and metabolizing enzymes. In this work, we compared the structural model of rat AMPKβ1 binding to β-cyclodextrin with AKINβγ and AKINβ2. In our bioinformatic analysis, we discovered that the two-tryptophan residues that are important for interaction with the carbohydrate (W100 and W133 in rat AMPKβ1) were conserved in AKINβγ and AKINβ2, but lysine (K126) and asparagine (N150) were present only in AKINβγ. In AKINβ1 only the W100 and the K126 were conserved. We identified an insertion of three amino acids in AKINβγ not present in AKINβ2, AKINβ1 and rat AMPKβ1, forming an extended loop next to one of the conserved tryptophans. AKINβγ and AKINβ2 expressed as recombinant proteins bound starch *in vitro*, but when using a mixture of amylose/amylopectin (A/A), AKINβγ showed higher association. Subcellular localization indicated that AKINβγ was present in the cytosol and in the chloroplasts, whereas AKINβ2 was mainly associated with the outside of the chloroplasts. Activity assays that included a mixture of A/A did not inhibit kinase activity, which correlates with the finding that SnRK1 bound to the A/A mixture was active. In contrast, addition of starch inhibited the kinase activity and proteins associated to this carbohydrate showed little activity. Interestingly, only the AKIN10, AKIN11, and AKINβγ subunits were present in the A/A complex or found associated with the starch, suggesting that a dimer between either of the catalytic subunits and AKINβγ could form an active complex.

## Materials and methods

### Plant material

Arabidopsis (*Arabidopsis thaliana*) plants were grown in a growth chamber at 21 to 23°C under short-day conditions (8 h of light/16 h of dark) at a light intensity of 120 μmol photons m^−2^ s^−2^ (fluorescent bulbs). Leaves from two-week old plants were used for the kinase assays and the subcellular localization experiments.

### Purification of recombinant proteins

The cDNA sequence corresponding to AKINβγ (At1g09020) was amplified with Pfu polymerase and cloned into the pGEM T-easy vector (Promega, WI USA). The plasmid was digested with *Nco*I and *Not*I enzymes and the released fragment was purified and cloned into a pET28b+ expression vector (Merck, Millipore USA). The cDNA sequence of AKINβ2 (At4g16360) was also amplified with Pfu polymerase and cloned directly into the pET101/D-TOPO vector (Invitrogen). The resulting expression vectors were transformed into the *Escherichia coli* BL21 (RIL) strain, and the recombinant proteins were induced and purified through a Ni-NTA column as indicated by the manufacturer (Qiagen, Mexico).

### Sequence and structural alignments

The sequences for AKINβγ, AKINβ1, and AKINβ2 were obtained from the Arabidopsis information resource (TAIR). The sequence alignment was carried out with the Multiple Alignment with Fast Fourier Transform: MAFFT program (version 7.0) through the online server (http://www.ebi.ac.uk/Tools/msa/mafft/). The parameters selected were the Blosum62 matrix, a gap open penalty of 1.53 and a gap extension penalty of 0.123.

### Structural models

The structural models for AKINβ2 and AKINβγ were obtained using the online version of the I-TASSER algorithm, which predicts the 3D structure with high accuracy by scoring multiple threading templates. Five models were obtained for each protein, and the best model was selected according to a C score close to −2.5 in combination with a TM score of approximately 0.45 for both proteins. The structural alignments between the 3D models and the structure of the homologous rat AMPKβ1 protein were obtained with the combinatorial extension method for structural superposition implemented in the pymol algorithm (version 1.5). For the best models, the 3D structure was structurally minimized with the Yasara algorithm (version 12.1.19). To analyze the carbohydrate-protein interactions, a docking approach was utilized between β-cyclodextrin and the protein. The structure of β-cyclodextrin was obtained from rat AMPKβ1 (PDB ID: IZ0M). The force field parameters for β-cyclodextrin were obtained through a module of the Yasara software that optimizes the structure using semi-empirical quantum chemistry and calculate the AM1 charges.

### Extraction of soluble protein from arabidopsis leaves

The total soluble protein was extracted in homogenization buffer containing 100 mM Tricine-NaOH (pH 8), 5 mM dithiothreitol (DTT), 0.5 mM ethyleneglycoltetraacetic acid (EGTA), 0.5 mM ethylenediaminetetraacetic acid (EDTA), and 1 mM benzamidine. Prior to homogenization, 1 mM phenylmethylsulphonyl fluoride (PMSF), 1× protease inhibitor cocktail (Sigma, Mexico), 1× phosphatase inhibitors (50 mM sodium fluoride, 25 mM β-glycerolphosphate, 10 mM sodium pyrophosphate, and 2 mM sodium orthovanadate) and insoluble polyvinylpyrrolidone (2% w/v) were added. The homogenate was transferred to microfuge tubes, and the insoluble material was removed by centrifugation (13000 × *g*) at 4°C for 20 min. The supernatant was desalted using an NAP-5 column (GE Healthcare, PA USA) that was pre-equilibrated in homogenization buffer. The desalted soluble protein was stored at −80°C until further use.

### Starch purification and binding assays

Arabidopsis plants were grown under short day conditions (8 h of light/16 h of dark) and leaves were harvested at the end of the light period. The starch granules were isolated from 15 g of Arabidopsis rosette leaves. The plant material was homogenized with a mortar and pestle and 3 volumes of isolation buffer containing 100 mM HEPES-KOH (pH 8), 1 mM EDTA, 5 mM DTT, 1 mM PMSF, and 0.05% Tween 20. The tissue lysate was filtered through two layers of cheesecloth and centrifuged for 5 min at 3000 × *g* at 4°C. The starch pellet was solubilized in isolation buffer and centrifuged at 9000 × *g* for 10 min through 95% Percoll (in 25 mM HEPES-KOH, pH 7). The pellet was washed several times with buffer until no green residue was observed and then washed with acetone. The starch granules were dried at 37°C overnight.

For the *in vitro* binding assays, 5 mg of starch or 10 mg of an amylose/amylopectin mixture (A/A, 70% amylose content, Megazyme, Ireland) were incubated with 5–10 μg of AKINβγ and AKINβ2 or 40 μg of the desalted soluble proteins in 50 mM Tris-HCl buffer, pH 7.5, for 30 min at 4°C, followed by centrifugation for 10 min at 5000 × *g*. The pellet was washed several times in the same buffer and resuspended in 20 μl of the sample buffer. The samples were subjected to SDS-PAGE, and the Western blot analysis was performed using AKINβγ- and AKINβ2-specific antibodies (Fragoso et al., 2009). Additionally, pellets containing the leaf protein-A/A and leaf protein-starch complexes were resuspended in 50 μl of 50 mM Tris-HCl, pH 7.5, and the kinase assays were performed using 15 μl of the suspension.

### Subcellular localization of AKINβγ and AKINβ

The leaves of the Arabidopsis plants were fixed and treated as described by Fragoso et al. (2009). Leaf sections were incubated with the primary rabbit AKINβγ and AKINβ2 antibodies (1:500 dilution) at 4°C overnight. These sections were then rinsed with PBS and incubated with a secondary goat anti-rabbit Alexa 568-fluorochrome-conjugated antibody for 4 h at 4°C. The sections were examined by confocal fluorescence microscopy using a FV1000 microscope (Olympus, Mexico).

### Chloroplast extraction and thermolysin treatment

The chloroplasts were isolated as described by Weigel and Glazebrook (2002) and resuspended in 100 μl of ice-cold 25 mM HEPES-KOH, pH 7.5, and 330 mM sorbitol buffer. The chloroplasts were transferred to a fresh 1.5-ml microcentrifuge tube, where 5 μ l of 2 mg/ml thermolysin was added and the mixture was incubated for 30 min on ice. The reaction was halted by the addition of 0.5 M EDTA to a final concentration of 10 mM, and the chloroplasts were re-isolated by centrifugation through a 40% Percoll cushion containing 50 mM HEPES-KOH, pH 7.5, 330 mM sorbitol and 5 mM EDTA. The thermolysin-treated and the untreated chloroplasts were stored at −80°C until further use.

### SnRK1 activity assays

The SnRK1 activity assays were conducted in a total volume of 25 μl at 30°C. The reaction mixture for each assay contained 40 mM HEPES, pH 7.5, 5 mM MgCl_2_, 200 μM ATP containing 12.5 kBq (γ−^33^P) ATP (PerkinElmer, MA USA), 200 μM of the AMARA peptide (AMARAASAAALARRR), 4 mM DTT, 0.5 μM okadaic acid and 1× protease inhibitor cocktail (Sigma, Mexico). In some activity assays, 0.4 mg of A/A or 0.5 mg of starch granules was included in the reaction mixture. The assay was then initiated by the addition of the extract containing the protein kinase. After 6 min, a 15 μL aliquot of the reaction mixture was transferred to a square (2 × 2 cm) piece of phosphocellulose paper (Whatman P81, Whatman), which was immediately immersed in 1% (v/v) phosphoric acid. The papers were washed three times with phosphoric acid, followed by acetone. The incorporation of ^33^P was quantified by liquid scintillation counting (ACSII Aqueous Counting Scintillant, Amersham) in a Beckman Scintillation Counter.

## Results

### Structural analysis

**Figure 1:**
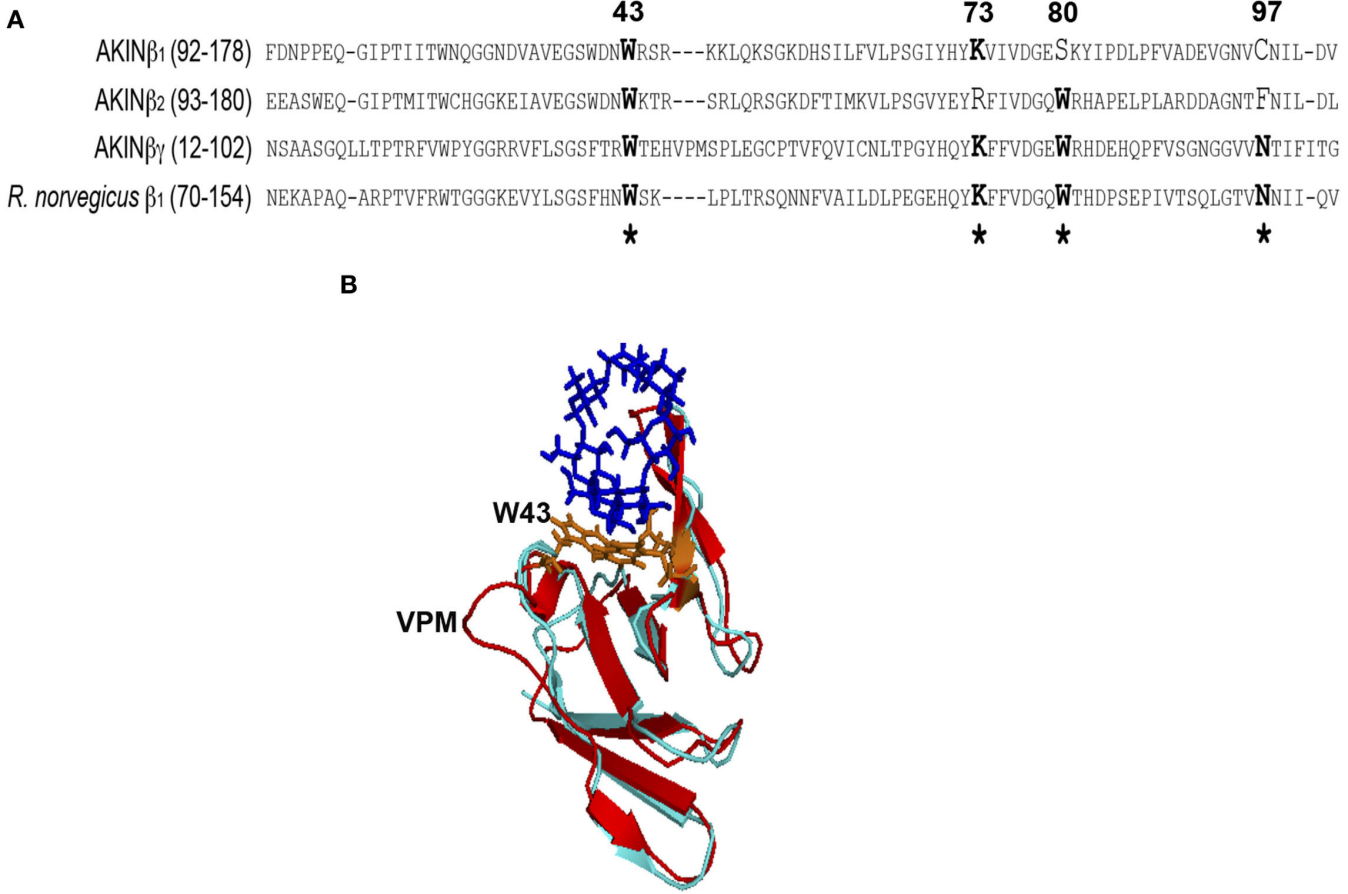
**Sequence and structural alignment**. **(A)** Amino acids 92–178 from AKINβ1, 93–180 from AKINβ2, 12–102 from AKINβγ and 70–154 from rat AMPKβ1 were included for the sequence alignment. The sequence identity with the Glycogen Binding Domain (GBD) of rat used to construct the structural model was 42%. **(B)** Lateral view of the structural alignment for the models generated with AKINβ2 (light blue) and AKINβγ (red) where the insertion of the three amino acids (VPM) is indicated. The structure of the β-ciclodextrin is showed in dark blue and the conserved tryptophans in orange. ^*^Indicates the conserved critical residues for protein-carbohydrate interaction.

The sequence and structural analysis of the regulatory β subunits from AMPK and SNF1, the animal and yeast homologs of SnRK1, indicated that the GBD recognizes and binds glycogen (Polekhina et al., 2003, 2005; Amodeo et al., 2007) and that this binding inhibits AMPK activity (McBride et al., 2009). In certain alignments, the plant β subunits exhibited the conserved amino acid residues that are important for interaction with the carbohydrate (McBride et al., 2009; Janecek et al., 2011), but no further studies have been performed. To evaluate the importance of this domain for the plant proteins, the sequence and structural alignments was carried out to compare the SBD of the AKINβγ and AKINβ2 subunits with the GBD of rat AMPKβ1. We did not include AKINβ 3 in our bioinformatic analysis because it does not have a GBD. The predicted structural model showed similar topology for AKINβγ and AKINβ2 compared to the GBD of AMPKβ1 (Figure 1). The sequence alignment for the SBD of AKINβγ and AKINβ2 with AMPKβ1 indicated sequence conservation for the binding site residues (Figure 1A). Based on the AKINβγ sequence, the tryptophan residues at positions 43 and 80 are conserved for both proteins, but the lysine at position 73 and the asparagine at position 97 are conserved only in AKINβγ. Furthermore, the sequence alignment indicated an insertion of three residues (VPM) in AKINβγ compared to AKINβ2 and AMPKβ1 (Figure 1A). This insertion creates an extra loop in the structural model that is adjacent to one of the tryptophan residues interacting directly with the carbohydrate (Figure 1B). In AKINβ1 only the W43 and the K73 were conserved inside the SBD, suggesting a different function.

### Binding of AKINβγ and AKINβ to starch and to amylose/amylopectin

**Figure 2:**
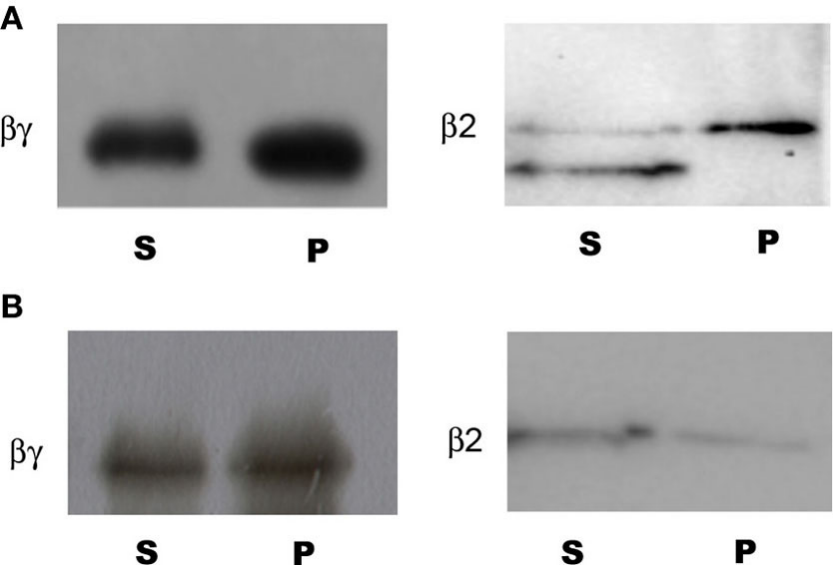
**Binding of AKINβγ and AKINβ2 to starch and A/A**. Samples of each protein were incubated with starch and A/A as described in the Materials and Methods section. The carbohydrate-protein complexes were collected by centrifugation and samples of the soluble fraction (S) and bound fraction (P) were separated by SDS-PAGE, and Western blot analysis was performed using protein-specific antibodies. **(A)** Protein-starch complexes. **(B)** Protein-A/A complexes.

The binding experiments using the AKINβγ and AKINβ2 recombinant proteins indicated that both proteins associated with the starch (Figure 2A). However, when using a mixture of A/A, AKINβγ interacted to the same extent, but AKINβ2 had a little association (Figure 2B).

### Subcellular localization of AKINβγ and AKINβ

**Figure 3:**
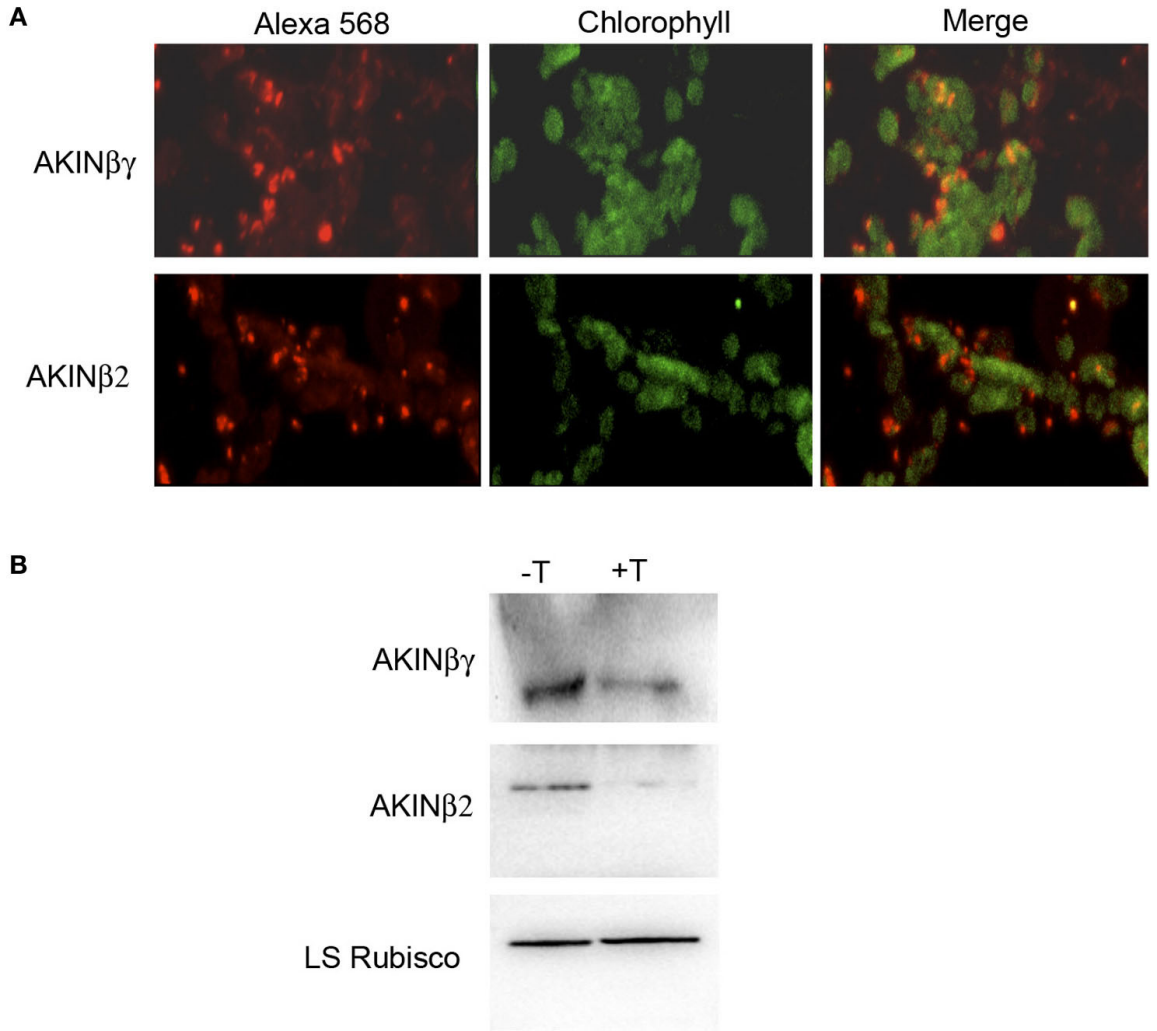
**Immunolocalization of AKINβγ and AKINβ2**. **(A)** Leaf sections incubated with AKINβγ- and AKINβ2-specific antibodies showing goat anti-rabbit Alexa 568-fluorochrome staining (red signal, 40×), chlorophyll (green signal, 40×) and overlaid images of the Alexa 568 fluorochrome and chlorophyll (40×). **(B)** The proteins extracted from the chloroplasts treated with (+T) or without (−T) thermolysin were analyzed by Western blot using AKINβγ- and AKINβ2-specific antibodies. The large subunit of Rubisco was used as a loading control.

To identify if there is colocalization of AKINβγ and AKINβ2 with starch, the leaf sections were analyzed using specific antibodies. Confocal fluorescence microscopy detected the AKINβγ signal in close contact with the chloroplasts, exhibiting a yellow color that indicates overlapping signal with the chlorophyll. For AKINβ2, the signal was detected in close proximity to the chloroplasts, but little yellow color was observed (Figure 3A). To determine if the proteins were inside the organelle, we isolated chloroplasts and treated them with thermolysin to eliminate all proteins interacting on the external surface. The proteins obtained from the protease-treated and untreated chloroplasts were subjected to Western blot analysis. The results revealed that AKINβγ was present in both the treated and untreated chloroplasts, indicating the presence of this protein inside and outside the organelle. In contrast, the majority of the AKINβ2 signal disappeared with protease treatment, suggesting that AKINβ2 interacts with the outer membrane of the chloroplasts and only a small fraction is inside (Figure 3B).

### Effect of A/A and starch granules on the SnRK1 activity

**Figure 4:**
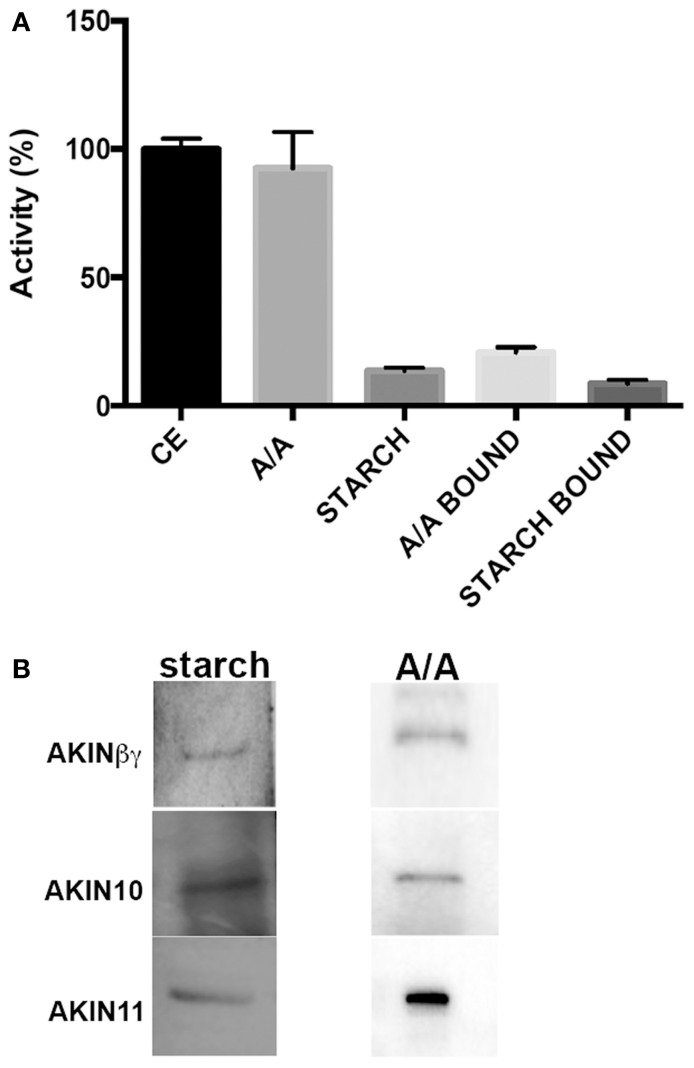
**Effect of A/A and starch on SnRK1 activity**. **(A)** SnRK1 kinase activity measured under normal conditions (CE), with 0.4 mg of A/A in the reaction mixture (A/A), with 0.5 mg of starch (STARCH) and using the protein-A/A complex (A/A bound) and the protein-starch complex (STARCH bound) as protein source. Specific activity for the control was 2.04 ± 0.08 nmol/min/mg. **(B)** Western blot analysis of the proteins extracted from the starch (starch) and from the A/A complexes (A/A).

To determine if the starch and the A/A mixture had an effect on SnRK1 activity, the protein obtained from Arabidopsis leaves was used to measure the SnRK1 activity. Assays containing A/A in the reaction mixture showed no effect on the activity in comparison with assays containing starch, which showed 85% of inhibition (Figure 4A). To evaluate if the proteins bound had kinase activity, we obtained the carbohydrate-protein complexes and used them as a protein source in the kinase assays. The results indicated that 20% of the total SnRK1 activity co-purified with the A/A pellet, and less than 10% of the total activity co-purified with starch (Figure 4A). To identify the SnRK1 subunits that co-precipitated with the carbohydrates, we incubated the protein-carbohydrate complex with SDS sample buffer. The proteins were separated by SDS-PAGE and transferred to a PVDF membrane. Western blot analysis identified AKIN10, AKIN11 and AKINβγ as the proteins associated with A/A (Figure 4B). There was no indication of any other SnRK1 subunits. Interestingly, Western blot analysis of the proteins associated with the starch using antibodies against all SnRK1 subunits identified the same subunits (Figure 4B).

## Discussion

### Structural analysis

Starch binding domains (SBDs) are motifs of approximately 100 amino acids present in several microbial amylolytic enzymes. These motifs are included in 10 Carbohydrate Binding Module (CBM) families (Janecek et al., 2011). The β subunits from AMPK and its homologs in yeast and plants belong to the CBM48 family. Because several initial representatives of this family had a role in glycogen metabolism, it was suggested to name them GBDs instead of SBDs (Janecek et al., 2011). Nevertheless, in the case of plant subunits, we propose a return to the initial classification to label them SBDs, reflecting their ability to bind starch.

The crystal structure of rat β1GBD in complex with β-cyclodextrin contained five important residues for carbohydrate binding, including two tryptophans, one lysine, one asparagine, and one leucine (Polekhina et al., 2005). Most of these residues are conserved in AKINβγ especially, and a model comparing the structure of the three proteins was highly similar in the overall topology. There was, however, an important difference between the plant AKINβγ and the two other proteins. This difference was in the region contiguous to tryptophan 43 (based on the AKINβγ sequence) where there was an insertion of three amino acids (VPM). It was previously proposed that this insertion might create a flatter surface that can associate with starch better than glycogen, which is important for the plant proteins (Polekhina et al., 2005). The same insertion was observed in maize AKINβγ, although no functional implication was suggested (López-Paz et al., 2009). Although AKINβ1 had only two of the conserved amino acids for carbohydrate binding, functional characterization should be performed to evaluate the ability to bind starch or other soluble sugars. Experiments to evaluate this possibility are currently ongoing.

### Binding of AKINβγ and AKINβ to starch and to amylose/amylopectin

The AKINβγ and AKINβ2 proteins exhibited important differences in their ability to bind starch and the A/A mixture. A reason for this differential binding could be the degree of branching found in the starch granules in contrast to the A/A mixture, which contains a 70% of amylose. A similar observation was made for AMPK, which differentially bound glycogen preparations obtained from various sources. McBride et al. (2009) concluded that the degree of starch branching affects the protein-carbohydrate interaction, thus indicating that the different glycogen preparations had a heterogeneous structure. In addition, the extra loop observed in AKINβγ could have influence on the interaction with some carbohydrates. For AMPKβ2, a threonine insertion contiguous to tryptophan 100 (equivalent to tryptophan 43 in the AKINβγ sequence) modified the affinity toward certain types of carbohydrates in comparison with AMPKβ1, which does not have this amino acid (Koay et al., 2010). The same could be applicable for the plant subunits, and modifications of this region will provide additional clues regarding its function.

### AKINβγ and AKINβ localization

The prediction of subcellular location by some computational resources indicated that AKINβγ and AKINβ2 were chloroplast proteins. Subcellular localization studies showed that both proteins were associated with chloroplasts, but in the case of AKINβ2, this association was predominantly on the outside. This implies that AKINβ2 will be functionally associated with the starch only in a minor proportion. It was previously suggested that the cytosolic β subunits in plants could bind small carbohydrates such as sucrose or trehalose 6-P (T6P), which are important for cell signaling (Polekhina et al., 2005). Interestingly, the evaluation of AKINβ2 binding with T6P and glucose 6-phosphate (G6P), inhibitors of SnRK1 activity (Nunes et al., 2013b), indicated that neither interacted with the protein (Coello, in preparation). However, we cannot rule out the possibility that both inhibitors could bind the AKINβ2 when in the heterotrimer conformation. The experiments to evaluate this possibility are ongoing. Additionally, it is known that as a product of starch degradation, maltose is exported to the cytosol and is metabolized by a glucosyltransfer reaction catalyzed by the cytosolic disproportionating enzyme, DPE2 (Chia et al., 2004). DPE2 transfer one glucose unit from maltose to an acceptor, a water-soluble heteroglycans (SHGs) and releases the other glucose molecule (Fettke et al., 2005a,b). Because the amount of glucose present in the heteroglycan is very low considering the amount of maltose released during starch degradation, it has been proposed that SHGs serves as a short-term acceptor for glucose in the cytosol (Chia et al., 2004). Experiments should be done to determine if AKINβ2 has affinity for this polymer and serves as a carbohydrate sensor in the cytosol. Because AKINβγ is inside the chloroplast and is recognized in the SnRK1 subunits that co-purified with the starch granules, it might participate in the regulation of starch metabolism by SnRK1 (McKibbin et al., 2006; Baena-González et al., 2007; Fragoso et al., 2009).

### SnRK1 activity is inhibited by starch

The GBD in the AMPK and SNF1 complexes is recognized as a regulatory domain, affecting kinase activity (Momcilovic et al., 2008; McBride et al., 2009; Mangat et al., 2010). For SnRK1, the presence of A/A did not modify its activity. Furthermore, SnRK1 activity was detected after binding the soluble proteins from Arabidopsis leaves to A/A in the protein-carbohydrate complex, strongly suggesting that SnRK1 is in an active conformation when it is bound to the carbohydrate. In contrast, the presence of starch strongly inhibited SnRK1 activity, indicating that after binding, the enzyme lost its active conformation and is unable to phosphorylate the AMARA peptide. We identified both catalytic subunits (AKIN10 and AKIN11) and AKINβγ in the protein-carbohydrate complex, but were unable to recognize AKINβ2, AKINβ1, and AKINγ. These results strongly suggest that a dimer form of AKINβγ and any of the catalytic subunits may have kinase activity and could be associated to the starch granules. We hypothesize that this heterodimer regulates the activity of some starch binding enzymes.

## Author contributions

Conceived and designed the experiments: Patricia Coello, Eleazar Martínez-Barajas, Alejandro Sosa-Peinado. Performed the experiments: Alejandra Ávila-Castañeda, Natalia Gutiérrez-Granados, Ana Ruiz-Gayosso, and Alejandro Sosa-Peinado. Analyzed the data: Alejandro Sosa-Peinado, Eleazar Martínez-Barajas, and Patricia Coello. Wrote the paper: Alejandro Sosa-Peinado, Eleazar Martínez-Barajas, Patricia Coello.

### Conflict of interest statement

The authors declare that the research was conducted in the absence of any commercial or financial relationships that could be construed as a potential conflict of interest.
